# Sensation seeking correlates with increased white matter integrity of structures associated with visuospatial processing in healthy adults

**DOI:** 10.3389/fnins.2023.1267700

**Published:** 2023-10-26

**Authors:** Andrea Escelsior, Alberto Inuggi, Maria Bianca Amadeo, Batya Engel-Yeger, Alice Trabucco, Davide Esposito, Claudio Campus, Anna Bovio, Sara Comparini, Beatriz Pereira da Silva, Gianluca Serafini, Monica Gori, Mario Amore

**Affiliations:** ^1^Section of Psychiatry, Department of Neuroscience, Rehabilitation, Ophthalmology, Genetics, Maternal and Child Health (DINOGMI), University of Genoa, Genoa, Italy; ^2^IRCCS Ospedale Policlinico San Martino, Genoa, Italy; ^3^U-VIP Unit for Visually Impaired People, Fondazione Istituto Italiano di Tecnologia, Genoa, Italy; ^4^Faculty of Social Welfare and Health Sciences, Department of Occupational Therapy, University of Haifa, Haifa, Israel

**Keywords:** sensory profile, healthy adults, neuroimaging, sensation seeking, visuospatial processing, mental disorders, resilience factors

## Abstract

**Introduction:**

The ability to process sensory information is an essential adaptive function, and hyper- or hypo-sensitive maladaptive profiles of responses to environmental stimuli generate sensory processing disorders linked to cognitive, affective, and behavioral alterations. Consequently, assessing sensory processing profiles might help research the vulnerability and resilience to mental disorders. The research on neuroradiological correlates of the sensory processing profiles is mainly limited to the young-age population or neurodevelopmental disorders. So, this study aims to examine the structural MRI correlates of sensory profiles in a sample of typically developed adults.

**Methods:**

We investigated structural cortical thickness (CT) and white matter integrity, through Diffusion Tensor Imaging (DTI), correlates of Adolescent/Adult Sensory Profile (AASP) questionnaire subscales in 57 typical developing subjects (34F; mean age: 32.7 ± 9.3).

**Results:**

We found significant results only for the sensation seeking (STS) subscale. Positive and negative correlations emerged with fractional anisotropy (FA) and radial diffusivity (RD) in anterior thalamic radiation, optic radiation, superior longitudinal fasciculus, corpus callosum, and the cingulum bundle. No correlation between sensation seeking and whole brain cortical thickness was found.

**Discussion:**

Overall, our results suggest a positive correlation between sensation seeking and higher white matter structural integrity in those tracts mainly involved in visuospatial processing but no correlation with gray matter structure. The enhanced structural integrity associated with sensation seeking may reflect a neurobiological substrate linked to active research of sensory stimuli and resilience to major psychiatric disorders like schizophrenia, bipolar disorder, and depression.

## 1. Introduction

### 1.1. Sensory processing

Sensory processing is a process that requires gathering and interpreting exogenous and endogenous sensory information (e.g., taste, touch, smell, sight, hearing, equilibrium, pain). It is fundamental for life; it constantly drives interactions between an organism and its environment, allowing an adaptive response to ambient changes (Miller and Schaaf, 2008). Sensory processing alterations, expressed in hypo or hypersensitivity, profoundly affect daily functioning. The functional impact of these alterations defined the Sensory Processing Disorders (SPD) condition. Given the importance of sensory processing, it is not surprising that SPD is associated with cognitive and emotional alterations. Thus, it may lead to behavioral alterations and be related to different mental disorders (Serafini et al., 2017a; Bowyer and Cahill, 2018; Harrison et al., 2019), their clinical severity, and burden of comorbidity (Engel-Yeger et al., 2016a, 2017; Serafini et al., 2017a). Despite the crucial importance of sensory processing in shaping normal and psychopathological behaviors, this topic is currently under investigated (Dunn et al., 2016). Indeed, the body of MRI research concerning sensory profiles is limited to few articles, mainly focused on young-aged, typically developed populations (Yoshimura et al., 2017; Shiotsu et al., 2021) and neurodevelopmental disorders (Owen et al., 2013; Ohta et al., 2020).

### 1.2. The Dunn’s model of sensory processing

The Dunn’s model provides a conceptual framework to interpret individual-specific behaviors as responses to different sensory processing patterns (Dunn, 1997). The model describes the interaction between the neurological threshold to sensory stimuli (low or high) and the individual’s behavioral strategy to deal with their neurological threshold, i.e., active or passive. The Adolescent/Adult Sensory Profile (AASP) questionnaire defines different sensory profiles in accordance with the individual neurological threshold and the behavioral strategies adopted. The term “neurological threshold” indicates an individual’s intrinsic sensitivity to sensory stimuli, determining the intensity of sensory input requisite for perceptual awareness or response. Individuals characterized by a lower sensory threshold typically exhibit heightened perceptual acuity, registering and responding to sensory inputs more rapidly. Conversely, those with an elevated threshold exhibit diminished responsiveness, potentially failing to discern stimuli that are perceptible to others. The neurological threshold to sensory stimuli was assessed through items that measure the need for higher intensity sensory stimuli, or even absence of response (e.g., “I hum, whistle, sing, or make other noises,” “I do not notice when my name is called”). The behavioral response continuum indicates the individual tendency to adopt passive or active strategies in response to their environments, according to one’s neurological threshold. Regarding behavioral response, people with passive tendencies tend to adopt an internalizing modality of response to sensory stimuli, characterized by reduced actions aimed at modifying the environment to adapt to one’s sensory pattern, whereas individuals with active tendency actively control the type and amount of sensory input in their environments (e.g., “I use strategies to drown out sound for example, close the doors, cover my ears, wear ear plugs,” “I stay away from crowds”) (Metz et al., 2019). The Dunn’s four-quadrant model of sensory profile characterizes the individual neurological threshold and the behavioral response, through questions that cover the auditory, visual, tactile, gustatory, olfactory, and vestibular/proprioceptive sensory modalities, thus distinguishing four different profiles (Ben-Avi et al., 2012). The sensory profiles are: low registration, sensation seeking (STS), sensory sensitivity and sensation avoidance (Dunn, 1997). Specifically, *low registration* subjects have a reduced ability to perceive sensory stimuli due to a higher neurological threshold and a passive strategy, meaning that they do not actively seek rich sensory input that reaches their threshold. Subjects with *sensory sensitivity* have a low neurological threshold and a passive strategy, meaning they do not actively limit their exposure to unpleasant sensory stimuli. *Sensation-avoiding* subjects have a low neurological threshold and active behavioral self-regulation strategies; they avoid overwhelming sensory input and follow routine habits (Dunn, 1997). Finally, *sensation seekers* have a higher neurological threshold and active behavioral self-regulation strategies. They actively search for intense stimuli like places with bright lights, colors, and sounds (as in parties and malls). Sensation seekers are often characterized by a tendency to impulsive decision-making processes (Engel-Yeger and Dunn, 2011). Furthermore, this profile is associated with increased motor behaviors (Serafini et al., 2017b), attachment security (Jerome and Liss, 2004), extroversion and reduced interpersonal boundaries (Ben-Avi et al., 2012). Significantly, a stronger tendency towards sensation seeking has been linked to a stress-resilient phenotype characterized by a stable personality disposition (Norbury et al., 2016), and lower incidence of major psychiatric disorders such as psychosis onset (Parham et al., 2019), schizophrenia (Brown et al., 2002; Zhou et al., 2020), bipolar disorder (Engel-Yeger et al., 2016b), depressive symptoms (Miller et al., 2007) and clinical depression (Engel-Yeger et al., 2018), along with a more favorable response to antidepressant medications (Engel-Yeger et al., 2018). On the other hand, heightened sensitivity, avoidant, and low registration profiles resulted associated with risk of developing psychosis (Ortibus et al., 2012; Parham et al., 2019), schizophrenia (Machingura et al., 2022), bipolar disorder and depression (Engel-Yeger and Dunn, 2011; Engel-Yeger et al., 2016b, 2018; Serafini et al., 2017a). Despite the evidence corroborating its role in resilience to psychiatric disorders, sensation seeking patterns seem to confer a tendency toward impulsive behaviors, resulting in risky behaviors, gambling, and alcohol or drugs use (Roberti, 2004). This vulnerability appears to involve a combination of striatal high dopaminergic tone and a lower density of D2-type receptors (Norbury and Husain, 2015). Additionally, sensation seeking appears to correlate with borderline traits and is linked with heightened impulsivity, stereotypy, and irritability in developmental disorders (Gundogdu et al., 2023; Wei et al., 2023).

### 1.3. The neuroradiological correlates of sensory profiles

In recent years, researchers have addressed the topic of the neurobiological underpinnings of sensory processing. Few studies have explored the neural features of sensory processing with neuroimaging techniques able to assess grey and white matter integrity. In the healthy population, to our knowledge, only two studies explored the correlations between white matter (WM) alterations and sensory processing using the Adolescent/Adult Sensory Profile (AASP) questionnaire (Shiotsu et al., 2021). They reported a positive correlation between the Diffusion Tensor Imaging (DTI) microstructural alterations of the right uncinate and cingulate tracts and the sensory sensitivity and sensation avoiding scores in a sample of 84 healthy young adults (Shiotsu et al., 2021). More recently, a study of Nakagawa et al. on 99 healthy subjects aged 26.9 ± 6.9 years, reported significant correlations between right caudate mean diffusivity (MD) (*r* = 0.36; *p* < 0.001) and axial diffusivity (AD) (*r* = 0.37; *p* < 0.001), and tactile sensation avoiding (Nakagawa et al., 2023). Regarding gray matter integrity, Yoshimura and colleagues examined the differences in grey matter (GM) volume related to sensory profiles in 51 young, healthy volunteers (26 females) aged: 22.5 ± 4.5 (range: 19–43 years old). They reported a positive correlation between sensory sensitivity scores and the left dorsolateral PFC volume (Yoshimura et al., 2017). Among clinical populations, neuroimaging studies on sensory profiles consist of case–control studies on neurodevelopmental disorders. For example, Owen et al. reported among children with SPD a decreased FA and increased MD and RD, particularly in the posterior cingulate cortex (CC), corona radiata and thalamic radiations (Owen et al., 2013). Ohta and colleages reported that adults with autism spectrum disorder (ASD) and attention deficit hyperactivity disorder (ADHD) display a correlation between sensory sensitivity scores and RD in the posterior CC (Ohta et al., 2020). However, further research is needed to find objective measures that provide possible explanations for neural mechanisms underlying the linkage between sensory processing and psychopathology (Engel-Yeger et al., 2016b). To summarize, the relationship between sensory profiles and gray and white matter alterations is poorly investigated.

### 1.4. Aims of the study

Existing literature has identified a knowledge gap regarding the neuroradiological correlates of sensory profiles in healthy adults. Given this context, our study aimed to investigate the relationship between structural MRI findings and sensory profiles, using different techniques, in a cohort of typically developed adults. Understanding the neurobiological associations of sensory profiles is essential, as certain profiles may correlate with the onset of specific mental disorders. In contrast, profiles such as sensation seeking may be linked to resilience against major psychiatric disorders, which are characterized by extensive reductions in cortical thickness and white matter alterations. Based on this framework, we hypothesize a relationship between increased structural integrity and the sensation-seeking profile.

## 2. Methods

### 2.1. Participants and procedure

Sixty healthy controls were recruited with the following criteria. Inclusion: (1) age between 18 and 65 years, (2) willingness to participate in the study, (3) normal range for full-scale intelligence quotient scores measured with the Wechsler Adult Intelligence Scale, Third Edition, and (3) spoken language: Italian. Exclusion criteria: (1) history of psychiatric disorders, assessed through the Italian version of the Mini-International Neuropsychiatric Interview (Rossi et al., 2004), (2) first-degree familiarity with neuropsychiatric disorders, (3) presence of severe neurological and medical illnesses (e.g., vascular diseases, cancer), (4) alcohol and substance abuse during the previous three months and (5) the inability to undergo an MRI examination. In addition, sociodemographic data and medical and psychiatric status information were collected. Furthermore, each participant responds to the AASP, a 60-item self-report questionnaire used to assess sensory processing patterns (Shiotsu et al., 2021). The items are sorted equally into four traits reflecting Dunn’s model. The questionnaire requests the participants to indicate the frequency of their behavioral responses to sensory experiences in daily life on a five-point Likert scale. Norms exist for different age groups. Additionally, since AASP profile (e.g., sensation seeking) has been associated to increased impulsivity, stereotypy and irritability in developmental disorders (Gundogdu et al., 2023; Wei et al., 2023), subjects also underwent the Barratt Impulsiveness Scale, BIS-11 (Patton et al., 1995), evaluating their impulsivity levels. Its total score, BIS_T, was investigated together with AASP profiles. The study was granted the approval of the Ethical Committee of IRCCS Ospedale Policlinico San Martino, and all subjects gave informed consent. The study was performed in accordance with the relevant ethical guidelines and regulations.

### 2.2. MRI data recording

A 1.5-T GE scanner with a standard head coil was used. Foam pads were used to reduce head motion and scanner noise. Three-dimensional T1-weighted anatomical images were acquired in a sagittal orientation employing a 3D-SPGR sequence (TR/TE = 11.5/5 ms, I*R* = 500 ms, flip angle = 8 degree, FOV = 25.6 cm) with a resolution in-plane of 256×256 and slice thickness of 1 mm. DTI was acquired with a pure axial single-shot echo-planar imaging sequence. The diffusion sensitizing gradients were applied along 60 non-collinear directions (*b* = 1,000 s/mm^2^), together with 5 acquisitions without diffusion weighting (*b* = 0). Fifty-five contiguous axial slices were acquired with a slice thickness of 2.5 mm without a gap. The acquisition parameters were as follows: TR/TE = 13,750/93 ms; image matrix = 128×128; FOV = 24 cm; NEX = 1.

### 2.3. MRI data processing

This study aimed to correlate the four sensory profiles to cortical thickness (CT) and DTI metrics. CT correlates with AASP were investigated through a single whole-brain analysis. DTI metrics (FA, RD, AD, MD) were fitted and underwent a whole-brain tract-based spatial statistics (TBSS) analysis to define which tract reconstruct with tractography. The selected tracts were reconstructed, and their mean metrics were calculated.

#### 2.3.1. Anatomical data

3D T1-weighted MRI scans were converted to NIFTI format and resliced from sagittal to axial orientation. They were visually inspected, and their origin was set in correspondence with the anterior commissure. The following processes were then carried out with the Computational Analysis Toolbox (CAT, version 12.6) within SPM12 using MATLAB (version 2017b). All images were normalized using an affine followed by non-linear registration, corrected for bias field inhomogeneity, and then segmented into gray matter (GM), white matter (WM) and cerebrospinal fluid (CSF) components (Ashburner and Friston, 2005). Using six iterations, the Diffeomorphic Anatomic Registration Through Exponentiated Lie (DARTEL) algebra algorithm normalizes the segmented scans into a standard MNI space (Klein et al., 2009). Compared to the conventional algorithm, the DARTEL approach can provide more precise spatial normalization to the template (Matsuda et al., 2012). We performed a non-linear deformation on the normalized segmented images with the CAT12 toolbox as part of the modulation step. This modulation compares the absolute amounts of tissue corrected for individual differences in brain size (Cousijn et al., 2012). All segmented, modulated, and normalized GM and WM images were smoothed using 8-mm full-width-half-maximum Gaussian smoothing. The CT was evaluated according to the following methods. The surface extraction pipeline used topology correction (Yotter et al., 2011a), spherical mapping (Yotter et al., 2011b), estimation of local surface complexity, and local gyrification (Luders et al., 2006). Finally, cortex surfaces were smoothed (FWHM = 15 mm) and resampled to a 32 k mesh compatible with the Human Connectome Project (HCP). Individual values of mean WM and GM volumes were calculated in each ROI of the Neuromorphometrics atlas (labeled data provided by Neuromorphometrics Inc.). Mean CT values (mCT) within the ROIs defined in the a2000s atlas included in CAT were also calculated. In a parallel pipeline, following the canonical FSL anatomical one, the axial-reoriented T1 images underwent bias field correction, skull-stripping, and non-linear co-registration to the standard MNI template. The resulting transformations were later used to normalize DTI and rs-fMRI data.

#### 2.3.2. DTI data

The diffusion-weighted data were skull-stripped using the Brain Extraction Tool implemented in FSLv6.0^1^ and then corrected for distortions caused by eddy currents and movements. The diffusion tensor (DT) was estimated on a voxel-by-voxel basis using the DTIfit toolbox, part of the FMRIB Diffusion Toolbox within FSL, to obtain FA, MD, AD and RD maps, the latter obtained by averaging L2 and L3 images. A bedpostX processing was done on eddy-current corrected images to allow probabilistic tractography analysis later.

### 2.4. TBSS analysis

A TBSS analysis was performed on the whole group. Individual FA images of all subjects were non-linearly registered to a standard fractional anisotropy template.^2^ We did not create a study-specific skeleton template, but we non-linearly reported each subject’s fractional anisotropy map to the FMRIB58 skeleton (parameter –T in the tbss_3_postreg script). This was done to better segment our results with the xTRACT atlas, as described later. The same operations were subsequently applied to the individual mean, axial, and radial diffusivity images using the previously calculated transformation. Voxelwise cross-subject statistics were then applied to these data.

#### 2.4.1. TBSS results segmentation

TBSS results were segmented according to the xTRACT atlas. To do this, we first created a skeletonized version of each xTRACT’s tract in the standard space by masking the FMRIB58 skeleton with each volume of the xTRACT-tract-atlases-maxprob5-1 mm image. The number of voxels composing each skeletonized xTRACT atlas tracts was calculated. Then, for each of these tracts, we calculated how much of its extension (coverage %) was included in the TBSS results image. For each tract and DTI measure, the mean values of the significant voxels were calculated and then correlated with the AASP score of interest to exclude any outlier subject. The CC region, not present in the xTRACT atlas, was obtained from the “Atlas of Human Brain Connections”.^3^

### 2.5. Tractography of tracts of interest

In those tracts where more than 10% of the voxels were significant, an automated xTRACT analysis, using its default settings, was performed in the native space of each subject. Mean tract values, averaging all the voxels belonging to each tract, were calculated. Individual mean values within CC were calculated in the FMRIB58 space considering all the 31,704 voxels of such tract. Mean tract values were then correlated to AASP score of interest.

### 2.6. Statistical analysis

#### 2.6.1. Demographic and questionnaires

Data normality was verified with the Shapiro-Wilik test. Outliers’ values were detected and removed according to the Inter-Quartile Range (IQR) method (Krzywinski and Altman, 2014) using a scale factor of 2, roughly corresponding to 3.375 time the standard deviation in case data were normally distributed. The effect of gender, age and their interactions on each sensory profile and on BIS_T was investigated by running an ordinal regression model,^4^ a non-parametric factorial model that does not require neither homoscedasticity nor any linearity assumptions. For an easier data inspection, relationship between the four sensory profiles, the impulsivity score and age were expressed through a correlogram (corrplot function of corrplot R package).

#### 2.6.2. MRI whole-brain data

Regardless of the specific implementation of either FSL or SPM software, the same General Linear Model, composed by individuals’ four AASP profiles, the BIS_T score, age and gender, was tested. All values were preliminary demeaned. Contrasts evaluated the positive and negative correlation of each AASP profile and BIS_T with the MRI measure (either CT or a DTI metrics), correcting for participant’s age and gender. In such a context, where each regressor competed to explain data variance, multiple comparison correction was done at the voxel level in each specific MRI analysis package. CT was performed with the multiple regression model of SPM using default parameters and correcting for multiple comparison with false discovery rate (FDR) criteria with *q* < 0.05. For TBSS group analysis, correlation between DTI metrics and AASP and BIS_T parameters was carried out with non-parametric permutation tests (5,000 permutations) and output maps were threshold-free cluster enhancement (TFCE) corrected using a significance threshold of *p* < 0.05.

#### 2.6.3. Correlation between tracts DTI metrics and AASP

Mean DTI metrics of both TBSS results tracts’ portions and individual tracts obtained through tractography underwent outliers’ detection and removal according to the Inter-Quartile Range (IQR) method using a scale factor of 2 and were correlated with the AASP scores using partial correlation analysis (using Spearman or Pearson method according to data normality) correcting for age and BIS_T. We reported in the manuscript only those tracts whose value of ps survived from a false discovery rate (FDR) correction for multiple comparison (*q*-value <0.05) and had an R-value over 0.3.

## 3. Results

After each MRI recording session, images were visually inspected checking for the presence of any artefacts. Two participants were excluded for excessive movements’ artefacts. One participant abandoned the MRI recording session for personal reasons. Finally, 57 subjects were investigated.

### 3.1. Sociodemographic characteristics

Thirty-four females and twenty-three males, typically developing right-handed subjects, aged 32.7 ± 9.3 years, participated in this study. Their sociodemographic characteristics are summarized in Table 1. Age was not distributed normally (Shapiro–Wilk test *p* < 0.001).

**Table 1:** Sociodemographic characteristics, impulsivity score and sensory profiles quadrant scores distribution of the sample.

Variable	Mean	SD	Range	
Number (*N*)	57			
Age	32.7	9.3	20–59	
Gender, female (male)	34 (23)			
Race, Caucasian	57			
Dominant hand (right)	57			
BMI Kg/m^2^	23.2	2.1	17.8–27.7	
Full scale IQ	102	13	87–132	
BIS Total	63.8	5.5	55–81	
Sensory Profile		*N*	%	Range
Low registration	Under norm	15	26.3	15–23
	Norm	32	56.1	24–35
	Above norm	10	17.5	36–75
Sensation Seeking	Under norm	19	33.3	15–42
	Norm	35	61.4	43–56
	Above norm	3	5.3	57–75
Sensory sensitivity	Under norm	10	17.5	15–25
	Norm	36	63.2	26–41
	Above norm	11	19.3	42–75
Sensation avoiding	Under norm	6	10.5	15–26
	Norm	40	70.2	27–41
	Above norm	11	19.3	42–75

Above norm, one standard deviation above AASP normal range; Norm, similar to AASP normal range; Under norm, one standard deviation below AASP normal range.

AASP, adolescent/adult sensory profile.

### 3.2. Questionnaires analysis

**Figure 1:**
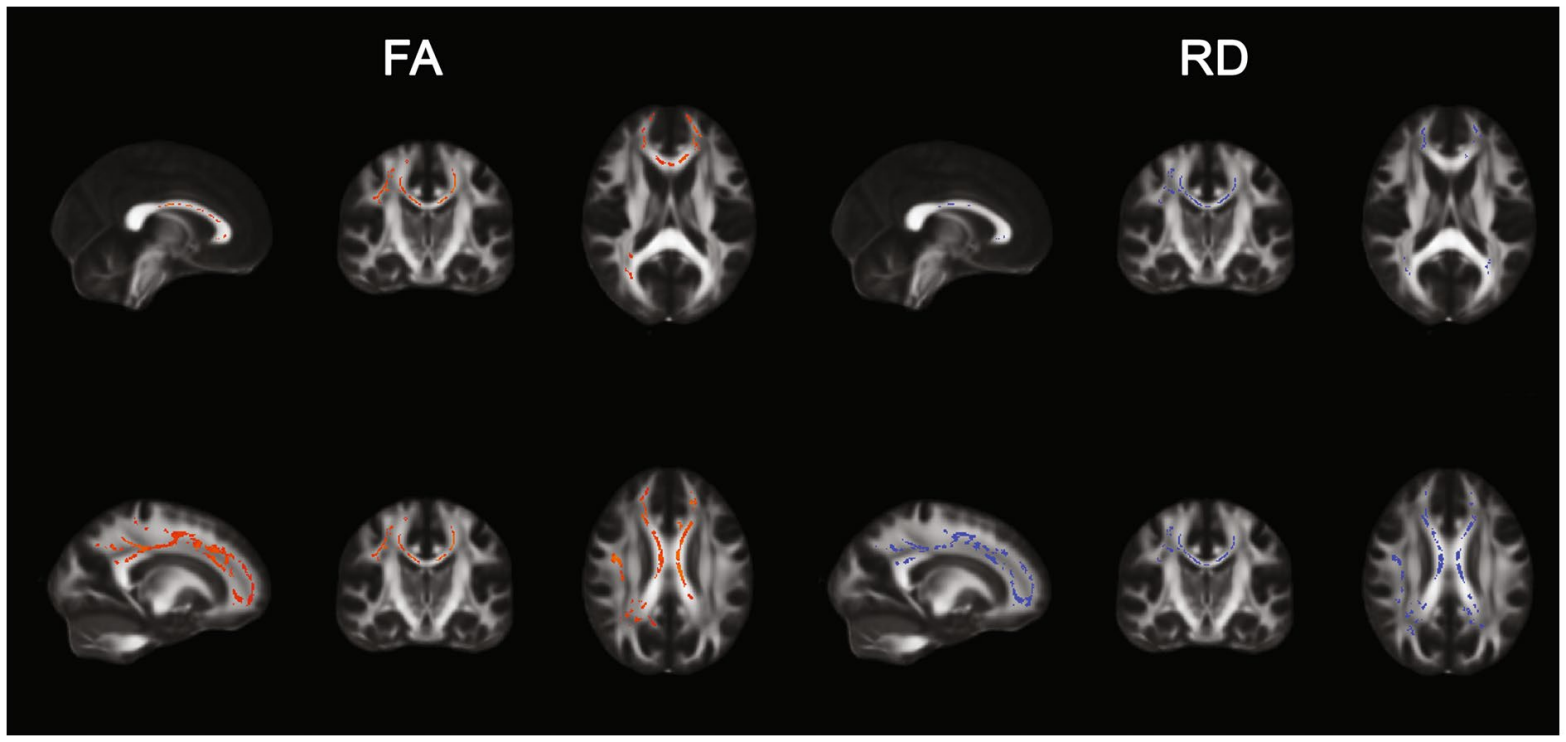
TBSS results. Positive (left) and negative (right) correlation between Sensation seeking (STS) and FA and RD.

Table 1 depicts the number and per cent of subjects with scores under the norm, in the normal range, and above the norm for each sensory profile. All sensory profiles score were normally distributed (Shapiro–Wilk test value of p are: sp_lr: *p* = 0.38, sp_sts: *p* = 0.16, sp_srs: *p* = 0.21, sp_sa: *p* = 0.11), while BIS_T not (*p* = 0.002). Factorial analysis did not find any significant effect of age and gender (nor their interaction) on AASP subscale scores and BIS_T. Table 2 reports the non-significant effect of gender over AASP and BIS_T. Since both age and BIS_T were not normally distributed, the correlogram, shown in Figure 1, was built running Spearman correlations. No correlation of either impulsivity and age with any of the AASP profiles could be observed.

**Table 2:** Gender effect on AASP and BIS_T scores (ORM model correcting for both age and gender).

	Male	Female	Gender effect
	(*n* = 23)	(*n* = 34)	value of *p*
Low registration	29.2 ± 7.2	28.9 ± 7.9	0.589
Sensation seeking	44.6 ± 5.2	44.7 ± 6.2	0.753
Sensory sensitivity	35.3 ± 6.6	33.1 ± 9	0.233
Sensation avoiding	35.5 ± 7.2	35.7 ± 9.3	0.801
BIS Total	63.8 ± 6.2	63.8 ± 5	0.703

### 3.3. Tract-based spatial statistics (TBSS analysis)

**Figure 2:**
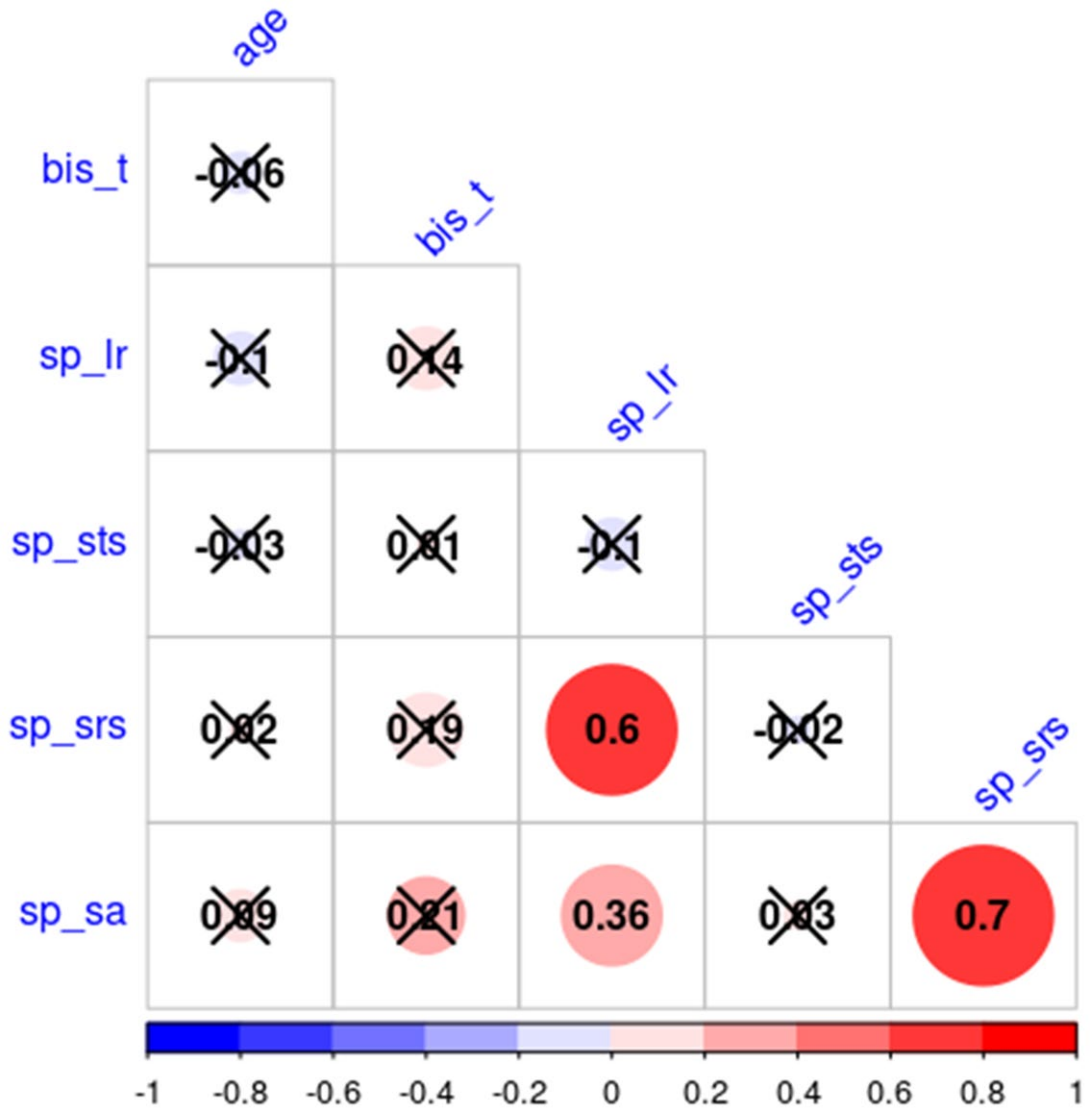
Correlogram results. Positive (red) and negative (blue) correlation between each AASP profile, age, and BIS_T. Non-significant correlations are marked with an X.

FA and RD resulted, respectively, positively and negatively correlated to the sensation seeking profile in a total of 16,104 and 12,043 voxels. Results are displayed in Figure 2. AD and MD were not correlated to any AASP dimension.

### 3.4. Segmentation of TBSS results with xTRACT atlas

We segmented both FA and RD significant TBSS maps with each xTRACT tract’s skeletonized version and found that 16 and 13 tracts had a fraction (higher than 10%) of their voxels’ values, respectively FA and RD, correlated with the STS score. The coverage percentages within each of these tracts are summarized in Table 3. Pearson correlation analysis confirmed their correlation with *STS* (columns tbss-R and tbss-P of Table 2). Through the automated xTRACT tractography analysis, we reconstructed these tracts (CC is not present in the xTRACT atlas) in each subject’s native space; we calculated their mean FA and RD and correlated it with STS, results are summarized in columns xTRACT-R and xTRACT-P of Table 3.

**Table 3:** TBSS and xTRACT results.

	FA					RD				
	TBSS			xTRACT		TBSS			xTRACT	
Tract	cov %	*R*	*q*	*R*	*q*	cov %	*R*	*q*	*R*	*q*
af_r	15.0	0.51	0.0002	n.s.	n.s	10.5	−0.26	0.054	n.s.	n.s
atr_l	15.1	0.5	<0.0001	0.36	0.022	14.47	−0.44	0.001	n.s.	n.s
atr_r	14.7	0.54	<0.0001	0.30	0.046	11.34	−0.45	0.001	−0.39	0.033
cbd_l	29.2	0.45	<0.001	0.35	0.022	20.13	−0.45	<0.001	−0.32	0.046
cbp_l	50.0	0.3	0.024	n.s.	n.s	12.0	−0.21	0.11	n.s.	n.s
cst_r	11.16	--	--	--	--	--	--	--	---	--
fa_l	11.4	0.38	0.0035	0.31	0.046	12.4	−0.41	0.003	−0.30	0.046
fa_r	14.1	0.43	<0.001	n.s.	n.s	11.12	−0.38	0.005	−0.31	0.046
fmi	43.9	0.49	<0.0001	0.30	0.040	35.9	−0.39	0.004	−0.32	0.046
mdlf_r	12.1	0.47	<0.001	0.4	0.020	--	--	--	---	--
or_r	17.3	0.38	0.0033	0.4	0.020	--	--	--	---	--
slf1_l	15.1	0.44	<0.001	n.s.	n.s.	14.0	−0.39	0.004	n.s.	n.s.
slf1_r	22.7	0.5	<0.0001	n.s.	n.s.	19.7	−0.51	<0.001	n.s.	n.s.
slf2_r	23.9	0.56	<0.0001	0.36	0.022	20.3	−0.47	<0.001	n.s.	n.s
slf3_r	13.7	0.53	<0.0001	0.38	0.022	--	--	--	---	--
cc	26.5	0.55	<0.0001	0.41	0.023	22.65	−0.50	<0.001	−0.36	0.021
str_r	--	--	--	--	--	10.4	−0.3	0.027	n.s.	n.s

Correlations between sensation seeking and DTI metrics: FA (left) and RD (right) of whole-brain TBSS and xTRACT based analyses. Cov %: percentage of voxels within each xTRACT tract that TBSS analysis found significantly correlated with sensation seeking. Correlation between sensation seeking and significant voxels of TBSS (tbss-R and tbss-P). Correlation between sensation seeking and all voxels of each xTRACT’s tract (xTRACT-R and xTRACT-P). All correlations’ *p*-values were corrected with FDR criteria and the adjusted *q*-values were reported.

*R*, regression coefficient; *q*, statistical significance after FDR; n.s., not significant.

atr, anterior thalamic radiation; cc, corpus callosum; cbd, cingulum subsection: dorsal; cbp cingulum subsection: peri-genual; cst, corticospinal tract; fa, frontal aslant tract; fmi, forceps minor; l, left; mdlf, middle longitudinal fasciculus; or, optic radiation; r, right; slf, superior longitudinal fasciculus; str, superior thalamic radiation.

### 3.5. CT whole-brain analysis

Whole-brain CT analysis did not reveal any significant vertex correlated to any sensory profile dimension.

## 4. Discussion

To the best of our knowledge, this is the first study evaluating sensory profiles patterns using both white and gray matter MRI measures in healthy adults. The study’s results showed a strong correlation between the AASP sensation seeking subscale score and several WM tracts involved in visuospatial sensory processing and affective regulation. More precisely, we found a positive and a negative correlation with both FA and RD in anterior thalamic radiation (ATR), optic radiation (OR), superior longitudinal fasciculus (SLF), CC, and the dorsal part of the cingulum bundle (dCB). We did not find significant correlations between AASP dimensions and cortical thickness. The present study will discuss the correlation among sensory profiles and tracts’ mean metrics rather than voxel-wise changes. In fact, albeit damages in white matter tracts in neurodegenerative diseases (e.g., multiple sclerosis) are often focal and sparse, we hypothesize that possible relations between tracts and AASP dimensions are related to a different development of the entire tract rather than the random occurrence of focal changes in some of its points.

### 4.1. Tracts with enhanced white matter integrity among sensation seekers

As previously reported, our results highlight the correlation between better integrity of the WM in multisensory and affective brain regions with sensation seeking scores. The optic radiation (OR) is a tract implicated in low-level simple visual processing (Winston et al., 2011), conveying visual inputs mainly from the magnocellular and parvocellular pathways, which project the visual dorsal and the ventral streams (Butler et al., 2005). The superior longitudinal (SLF) includes mainly frontoparietal and, to a lesser extent, frontotemporal connections (Kamali et al., 2014). Anatomically, the SLF comprises three subcomponents (SLF I, II, and III)(Schmahmann and Pandya, 2009). SLF II, and SLF III are involved in auditory processing in the dominant hemisphere and visuospatial processing in the right hemisphere (Barbeau et al., 2020); moreover, SLF III is implicated in facial emotion recognition (Nakajima et al., 2019). These WM connections mainly belong to the frontoparietal network and are crucial for sensory processing, cognitive functions, metacognition and empathy (Kamali et al., 2014). Regarding the anterior thalamic radiation (ATR) fibers, they mainly connect the mediodorsal thalamic nuclei with the PFC (Denier et al., 2020), the anterior thalamic nuclei and the anterior cingulate cortices (Zhou et al., 2003). The ATR mediates complex behavior planning (Mamah et al., 2010) and affective regulation (Coenen et al., 2012). Interestingly, children with SPD displayed cognitive and visuomotor control impairments linked to reduced FA in ATR and SLF (Brandes-Aitken et al., 2018). In children, sensation seeking, assessed through the UPPS-P scale, results negatively associated with the FA of the right ATR (Owens et al., 2020). The CC plays an essential role in the communication between the hemispheres (Tanaka-Arakawa et al., 2015), allowing the multidimensional representation of information and adaptative coordination of sensorimotor, affective and cognitive functions (Fenlon and Richards, 2015). The CC displays high plasticity in response to environmental stimuli (Tanaka-Arakawa et al., 2015; De León Reyes et al., 2020), and its structural integrity is associated with stress resilience (Galinowski et al., 2015), whereas reduced integrity is common among mental illnesses. Finally, the cingulum bundle (CB) is a vast WM structure connecting subcortical regions to the cingulate cortex and connecting parietal and medial temporal cortices (Bubb et al., 2018). The cingulum is a highly environment-sensitive structure with prolonged development. This reason might explain its crucial role in affective and neurocognitive differences between subjects (Bathelt et al., 2019) and its essential role in psychopathology (Bubb et al., 2018). Interestingly, the dorsal cingulate region is crucially involved in emotional self-control and dopamine-mediated reward prediction, providing adaptive decision-making responses to environmental conditions (Allman et al., 2001; Holroyd et al., 2004) and higher cognitive control (Bettcher et al., 2016).

### 4.2. Interpretation of DTI metrics change

In almost all tracts correlated with sensation seeking AASP dimension, a consistent pattern of DTI metrics was found; to a higher STS score, an increased FA and a reduced RD were observed. DTI metrics interpretation is centered on FA value which is a proxy of the degree of anisotropy present in the investigated white matter voxel. A higher anisotropy suggests the presence of well-formed fiber bundles that orient water molecules diffusion along their longitudinal directions. This orientation is determined not only by the hydrophobic myelinic structure that covers axons, anisotropy is in fact observed also in unmyelinated axons (Beaulieu and Allen, 1994; Kasprian et al., 2008), but also on axonal structure (Lebel et al., 2019). At least during development, FA and RD but not magnetization transfer ratio (MTR), a parameter known to be sensitive to myelin, varied with age, suggesting that axonal packing and membrane characteristics also play an important role in determining FA/RD changes (Moura et al., 2016). To try to disambiguate the concurrent effect of myelinization and axonal structure, AD and RD were also investigated. Reduction of the former has been mostly associated with axonal degeneration or fragmentation and reduction of the latter to myelinization degradation (Aung et al., 2013). Albeit these measures have been mainly used to interpret pathological degenerative processes within the white matter, we believe they might be used also in a physiological context like the present one. Since a reduced RD suggests a higher myelinization, we can argue that people with higher STS have more anisotropy (higher FA) due to more myelinated (lower RD) fibers. Recent studies have consistently shown that myelinization process is plastic and activity dependent (Fields, 2015; Noori et al., 2020), the stronger structural correlation between STS and the resulting tracts suggests that individuals with high STS might also have an increased functional connectivity among the areas they connect. Since higher myelinization contributes, together with axon inner diameter, to faster fibers’ conduction speed (Waxman, 1980; Salami et al., 2003), such increased connectivity would be mediated by higher conduction speed that, although *per-se* does not guarantee a higher brain connectivity efficiency, which is mainly a matter of synchronicity (Fields, 2015), can undoubtedly promote it.

### 4.3. Differences with previous studies and results discussion

Our results showed significant differences with the previous reports, which did not find significant correlations between DTI variables and the sensation seeking profile. A seminal study by Shiotsu and colleagues investigate the correlation between AASP profiles and the DTI variables in a sample of 84 healthy young adults (42 females) aged 24.5 ± 4.7 years (range: 19–39), finding significant correlations only between the sensory sensitivity and sensation avoiding scores and the right cingulum bundle axonal diffusivity and the sensation avoiding score and the right cingulum bundle mean diffusivity (Shiotsu et al., 2021). The discrepancies in the MRI findings between our study and that of Shiotsu and colleagues could be influenced by various factors. One notable consideration is that both studies operated with relatively small sample sizes, which might account for the differing results. Available literature highlights that *“the relationship between sensory processing and white matter microstructures remains largely unknown”* (Shiotsu et al., 2021). According to the Dunn’s model of sensory profiles, individuals with higher scoring in the AASP sensation seeking subscale constantly research environmental inputs and usually need to occupy their time (Dunn, 1997). Weak response and rapid habituation to stimuli bring these individuals to add sensory inputs to their routine to improve attention and vigilance towards the external signals, in contrast to other subjects, who tend to get distracted. The sensation seeking profile was associated with increased motor behaviors, enhanced motor impulsivity, decreased non-planning impulsivity (Serafini et al., 2017b), attachment security (Jerome and Liss, 2004), hyperactivity, extroversion, and reduced interpersonal boundaries (Ben-Avi et al., 2012). In this framework, our results might coherently reflect a possible association between higher white matter myelinization in bundles functionally involved in visuospatial processing and the subjective sensory experiences and the behaviors characteristics of the sensation seeking profile. Scarce literature regarding the impulsivity trait in typically developed population seems to support an association between increased FA and sensation seeking. A recent article on 390 adult individuals (mean age: 44.30 ± 18.59 years old) using the UPPS (urgency, premeditation, perseverance, and sensation seeking) scale, found a positive correlation between the auditory white matter tract FA and the impulsivity, especially in association with sensation seeking (Stansberry et al., 2022). Another article of Ikuta et al., reported on 143 healthy adults (mean age: 44.63 ± 17.15 years) a positive correlation between UPPS score and the FA of the accumbofrontal tract (Ikuta et al., 2018). However, according to our knowledge, the relationship between white matter integrity and sensory profiles is poorly investigated. Notably, although a single profile should not confer a unique vulnerability pattern associated with mental disorders, literature concordantly reported results indicating that the sensation seeking profile might be related to resilience to severe mental illnesses. Interestingly, low registration and lower tendency to seek sensory input are prevalent in psychiatric conditions, such as affective disorders (Serafini et al., 2017b). The available studies regarding sensory processing assessment in psychiatric disorders, in fact, concordantly suggest an association between resiliency to major psychiatric disorders and the sensory seeking profile. Different authors defined sensation seeking as “stress-resilient phenotype” (Miller et al., 2007), “a resilient pattern in the meaning of stable personality dispositions and psychiatric disorders” (Norbury et al., 2016), “a resilient factor” to major affective disorder (Engel-Yeger et al., 2016b), and, more recently, “a reliable measure to distinguish sensory responsiveness patterns between typically developed adolescents and schizophrenia patients” (Zhou et al., 2020). In line with these statements, evidence on different clinical populations, with neurodevelopmental (Bijlenga et al., 2017; Neufeld et al., 2021), mood (McCann et al., 1990; Miller et al., 2007; Engel-Yeger and Dunn, 2011; Serafini et al., 2016; Engel-Yeger et al., 2018) or psychotic disorders (Brown et al., 2002, 2020; Zhou et al., 2020; Gröhn et al., 2022), concordantly reported lower tendency to seek for sensory input, and individuals with sensory seeking experience reduced symptomatology (Serafini et al., 2016) and better quality of life (Engel-Yeger et al., 2016a). In the present study, no significant relationship was found between AASP dimensions and cortical thickness. Previous neuroimaging studies investigating AASP correlates did not include analyses on structural gray matter integrity, which makes not possible a direct comparison with our current findings. However, when considering white matter microstructure, it is plausible to suggest that the sensation seeker trait is not associated with an altered development of specific cortical areas. Instead, it may be linked to a more widespread increase in the anisotropy of brain tracts, possibly attributed to enhanced fiber myelination. This neurobiological basis of improved efficiency in brain structural connectivity could potentially explain why the sensation seeker trait has been considered a resilience factor against major psychiatric disorders. Nevertheless, further research is required to gain a better understanding of the complex relationship between sensation seeking behavior, brain connectivity, and its potential impact on mental health conditions.

### 4.4. Limitations

The present report has some limitations. Although this is an exploratory study, our cohort dimension is lower than many of the correlations studies nowadays presented. This likely affected the absence of results for gray matter volumes and cortical thickness. But meanwhile, it supports the importance and the solidity of our white matter tracts findings. MRI recordings were done with a 1.5 Tesla scanner. Although magnetic scanners of higher Tesla (e.g., 3 T) induce higher distortions in diffusion sequences, their doubled (compared with 1.5 T) SNR guarantees more minor variances in the estimated FA values (Alexander et al., 2006) and are thus preferable.

## 5. Conclusion

The sensation seeking profile is usually associated with a tendency to actively pursue novel sensory experiences and a tendency to impulsive decision-making processes. Here, we reported that such tendency in healthy subjects is strongly associated with a higher white matter structural integrity in those tracts interconnecting the areas mainly involved in visuospatial processing. Notably, cortical thickness could not be related to any AASP dimension. To explain this finding, further studies shall be carried out. In this framework, we may speculate that a possible neurobiological substrate of sensation seeking might be an increased structural integrity of several white matter tracts, fostering an improved widespread cortico-cortical connectivity, rather than modifications in specific grey matter regions.

## Data availability statement

The raw data supporting the conclusions of this article will be made available by the authors, without undue reservation.

## Ethics statement

The studies involving humans were approved by the Ethical Committee of IRCCS Ospedale Policlinico San Martino. The studies were conducted in accordance with the local legislation and institutional requirements. The participants provided their written informed consent to participate in this study.

## Author contributions

AE: Conceptualization, Formal analysis, Investigation, Writing – original draft, Writing – review & editing, Validation. AI: Conceptualization, Data curation, Formal analysis, Methodology, Writing – original draft, Writing – review & editing, Validation. MA: Validation, Writing – review & editing. BE-Y: Conceptualization, Writing – review & editing. AT: Data curation, Validation, Writing – review & editing. DE: Data curation, Validation, Writing – review & editing. CC: Data curation, Methodology, Writing – review & editing. AB: Data curation, Validation, Writing – review & editing. SC: Data curation, Writing – review & editing, Validation. BP: Writing – review & editing, Data curation, Funding acquisition. GS: Validation, Writing – review & editing, Conceptualization. MG: Supervision, Validation, Writing – review & editing. MA: Validation, Writing – review & editing, Project administration, Supervision.

## Footnotes

^1^http://fsl.fmrib.ox.ac.uk/fsl/fslwiki/

^2^http://fsl.fmrib.ox.ac.uk/fsl/fslwiki/FMRIB58_FA

^3^http://bcblab.com/BCB/Atlas_of_Human_Brain_Connections.html

^4^ORM, function of the rms R package, https://cran.r-project.org/web/packages/rms/index.html
